# Drug Policies Skyline during COVID-19 Pandemic

**DOI:** 10.3390/jcm10143117

**Published:** 2021-07-15

**Authors:** Serena Vita, Dora Forliano, Aldo De Luca, Alessia Beccacece, Luisa Marchioni, Emanuele Nicastri, on behalf of the COVID-19 INMI Study Group

**Affiliations:** 1 National Institute for Infectious Diseases, Lazzaro Spallanzani, IRCCS, Via Portuense 292, 00149 Rome, Italy; dora.forliano@inmi.it (D.F.); aldo.deluca@inmi.it (A.D.L.); alessia.beccacece@inmi.it (A.B.); luisa.marchioni@inmi.it (L.M.); emanuele.nicastri@inmi.it (E.N.); 2 Azienda Socio Sanitaria Territoriale Lariana, Via Napoleona 60, 22100 Como, Italy

**Keywords:** DDD, antivirals, steroids, low molecular weight heparin, hydroxicloroquine, COVID-19

## Abstract

The COVID-19 pandemic has produced an extraordinary care setting where physicians played, and continue to play, a critical role in containing viral spread and treating affected patients. Frontline workers have been receiving day-to-day new information about therapeutic advances. The purpose of the study is to analyse COVID-19 drug consumption trends in both acute and intensive care settings comparing Defined Daily Doses and the release of scientific clinical data from January to December 2020.

## 1. Introduction

At the end of January 2020, the novel Coronavirus Disease-2019 (COVID-19) epidemic spread to Italy. The resulting high rates of patients with pulmonary disease due to severe acute respiratory syndrome coronavirus-2 (SARS-CoV-2) infection overwhelmed the Italian health services. Management of inpatients was based on discordant or contradictory clinical protocols developed by the World Health Organization (WHO), national and international public health agencies, and infectious disease societies with very scarce medical evidence. Physicians involved in COVID-19 clinical management were confused and unsettled, waiting for information on therapeutic progresses and ready to eventually modify their standard of care in case of data from cases series, cohort studies, or clinical trials [1].

We analysed consumption trends of COVID drugs in a COVID-19 acute care setting (ACS) and in an intensive care setting (ICS) using drug Defined Daily Dose (DDD) according to the emergence of relevant scientific data from January 2020 to December 2020.

## 2. Materials and Methods

The National Institute for Infectious Diseases “Lazzaro Spallanzani” (INMI), is the reference COVID-19 hospital in Italy. The drug DDD is a unit of measurement of drug consumption, and is linked to the Anatomical Therapeutic and Chemical (ATC) classification system. The COVID drugs were unified by ATC to standardize the comparison of drug use in different health care environments. In the current analysis, we included antivirals (lopinavir/ritonavir, darunavir/ritonavir, and remdesevir), low-molecular-weight heparin (enoxaparin, fondaparinux), steroids (methylprednisolone, prednisolone, hydrocortisone, and dexamethasone), cloroquine and hydroxycloroquine, and tocilizumab (TCZ).

## 3. Results

**Figure 1:**
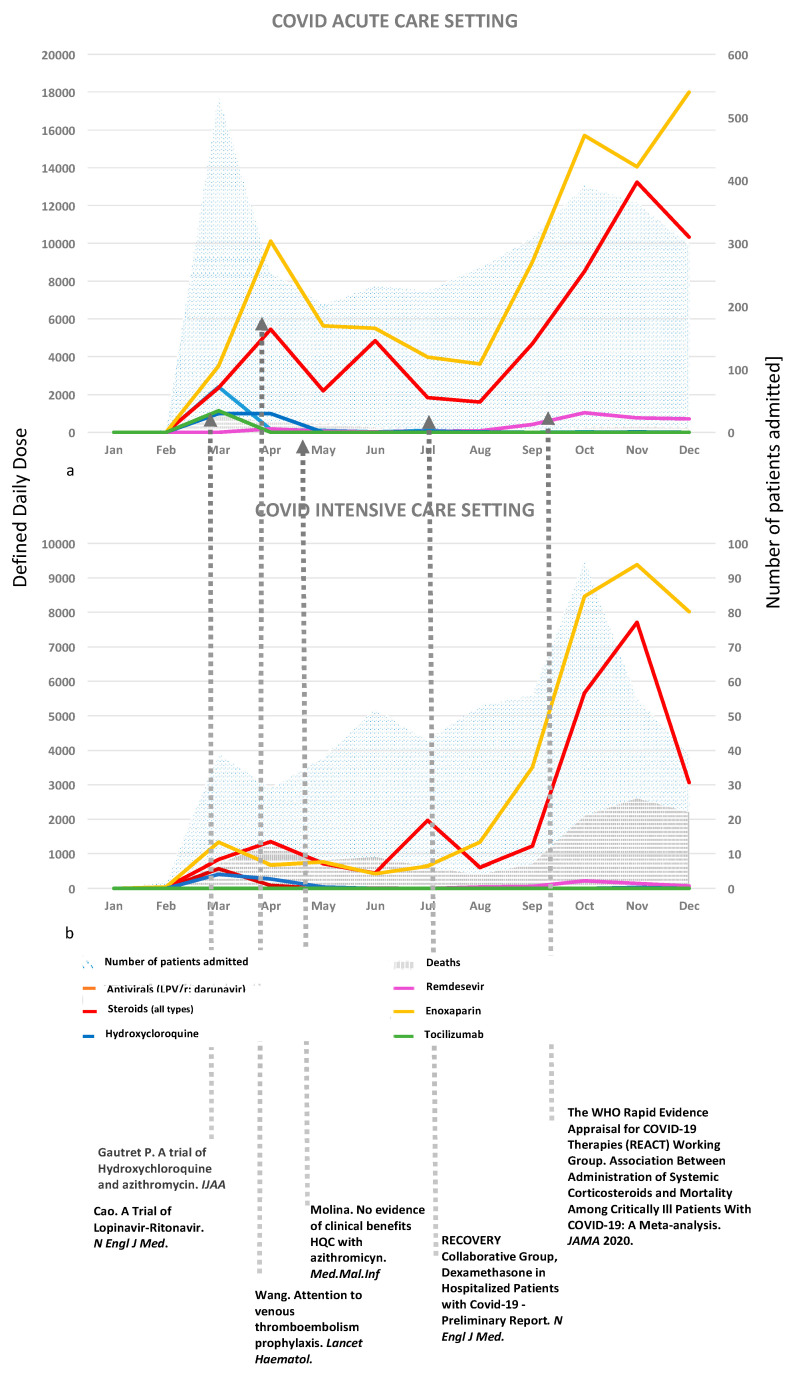
Admitted patients, deaths, and drug DDD trends in both ACS (**a**) and ICS (**b**) according to the emergence of relevant scientific data from January to December 2020.

In the year 2020, 3582 patients were admitted to INMI: 3081 patients (86%) were admitted in ACS, of whom 246 patients were referred to ICS and 103 patients died; 501 patients (14%) were directly admitted in ICS, of them 123 died. Drug DDD trends in both ACS and ICS are reported in Figure 1a,b.

**Figure 2:**
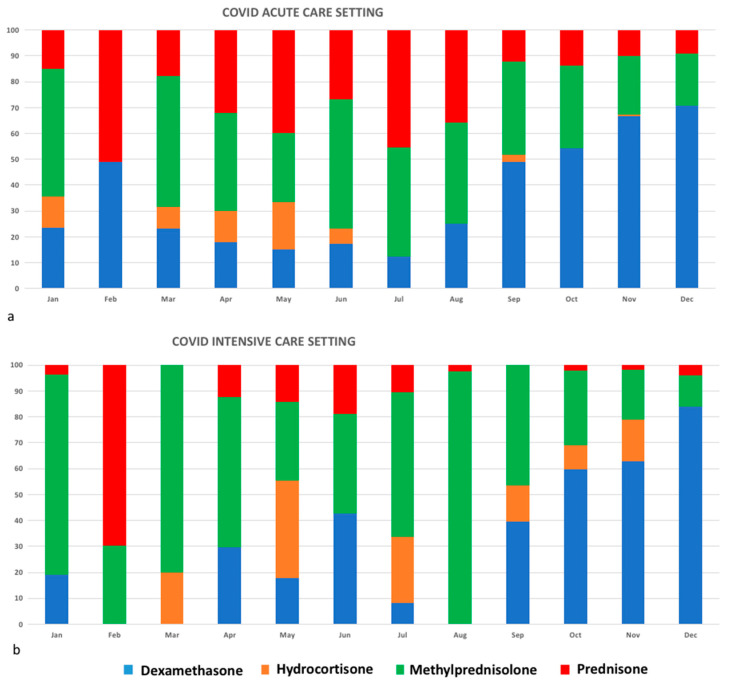
Different types of corticosteroids used in ACS (**a**) and ICS (**b**) from January to December 2020.

In ACS, non-COVID specific antiviral DDDs (lopinavir/ritonavir (LPV/r), darunavir/ritonavir) peaked in March 2020 immediately before the publication of unfavourable clinical results from randomized clinical trials (RCTs) conducted in China [2,3]. Remdesevir (RDV) was administered under compassionate use programmes until July 2020. Since August 2020, when it was approved for COVID-19 by the Italian Drug Agency (AIFA), increasing RDV DDDs were observed, with a peak of 700 DDDs in December 2020. Aminoquinolines such as Hydroxicloroquine (HCQ) were initially used in both ACS and ICS up to March 2020 (DDD 988 and 407, respectively), when a small clinical trial showed favourable virological and clinical results in patients treated with HCQ alone or in combination with azithromycin [4]. In May 2020, following the publication of a large observational study showing no clinical benefit [5], a progressive HCQ DDD drop-up was observed. Low-molecular-weight heparin (LMWH) DDDs steadily increased from March to December 2020 in both ACS and ICS since initial, and, thereafter confirmed, evidences of beneficial effects [6]. Tocilizumab was prescribed only in March in ACS, considering preliminary contradictory data on clinical outcome [7]. Corticosteroid use increased from February to December from 49 to 10,336 DDD in ACS, and from 415 to 3058 in ICS, with a peak immediately after the publication of the data from the recovery trial [8] in July 2020, and from the WHO living guidance on Corticosteroids for COVID-19 in September 2020 [9]. Dexamethasone and methylprednisolone were the most used corticosteroids in ACS (Figure 2a,b). Methylprednisolone was more commonly prescribed from February to July 2020 in both settings, whereas dexamethasone was used from August 2020 in ACS and from September 2020 in ICS.

## 4. Conclusions

According to PubMed (https://pubmed.ncbi.nlm.nih.gov/?term=covid-19, 13 June 2021), since the first report on 7 January, more than 150,000 papers on COVID-19 have been published.

Since the beginning of the pandemic, COVID-19 patients have been treated with lopinavir/ritonavir alone or, after the first reports of in vitro efficacy, in combination with HCQ and/or azithromycin, all drugs used in off label indications.

In our COVID referral centre, since March 2020, the use of these drugs (lopinavir/ritonavir, HCQ, and chloroquine) has steadily increased after the official approval of the drug repurposing policy released by AIFA [10]. In late May 2020, after clear evidence of likely prolongation of the QTc interval, and of unfavourable data on the use of HCQ, AIFA suspended the approval of HCQ [11]. A progressive decrease in HCQ DDDs was reported in our centre, as reported in other health care settings, such as France [1]. Finally, a study from the United States described a HQC drug shortage for patients affected by rheumatologic diseases between March and May 2020 [12]. It was followed by other reports worldwide of HCQ shortages and concerns about drug access for rheumatologic patients [13,14,15].

Preliminary data on RDV compassionate use were published on 10 April; RDV was associated with a clinical improvement in more than half of the cases with a 13% case fatality rate [16]. Later in April, Chinese data showed no significant clinical benefit in RDV-treated patients [17]. However, only a few hours later, Anthony Fauci officially presented favourable preliminary data in the time to recovery of RDV-treated patients from the Adaptive COVID Treatment trial [18]. In August, in line with the official approval by AIFA, the RDV DDDs increased in our centre.

During the first year of COVID-19 pandemic, only two drugs have been increasingly used: corticosteroids and LMWH. Their prescription has been based on preliminary and, then, confirmed clinical evidence [8,19]. Since September, a progressive increasing use of dexamethasone DDD among corticosteroids was reported in line with scientific evidence [8]. Unfortunately, due to the lack of specific scientific reports, no further considerations can be given.

Since the beginning of the pandemic, physicians dedicated to the clinical care of COVID-19 patients have been enriching their knowledge and modulating their clinical practice based on changing scientific evidence. In this context of urgent demand for combined pharmaceutical policies to fight the COVID-19 pandemic, all relevant scientific publications have been quickly shared throughout the scientific community. Many early reports of potentially effective—yet unconfirmed—anti-COVID drugs were emerging from limited case series, observational trials or post hoc analysis. Some of these preliminary findings were not confirmed in RCTs, rapid changes of treatment recommendations in national and international guidelines were observed, and disparate behaviours in combined pharmacological prescriptions were observed, even in COVID referral centres.

Today, physicians devoted to COVID clinical care have a more conservative attitude, waiting for more definitive and confirmatory data from evidence-based RCTs, before changing their therapeutic orientation.

## Data Availability

The data presented in this study are available on request from the corresponding author.
